# Combining vasculature disrupting agent and toll-like receptor 7/8 agonist for cancer therapy

**DOI:** 10.18632/oncotarget.14260

**Published:** 2016-12-27

**Authors:** Anushree Seth, Hyunseung Lee, Mi Young Cho, Cheongsoo Park, Sovannarith Korm, Joo-Yong Lee, Inpyo Choi, Yong Taik Lim, Kwan Soo Hong

**Affiliations:** ^1^ Bioimaging Research Team, Korea Basic Science Institute, Cheongju 28119, Republic of Korea; ^2^ Graduate School of Analytical Science and Technology, Chungnam National University, Daejeon 34134, Republic of Korea; ^3^ Immunotherapy Convergence Research Center, Korea Research Institute of Bioscience and Biotechnology, Daejeon 34141, Korea; ^4^ SKKU Advanced Institute of Nanotechnology (SAINT), Sungkyunkwan University, Suwon 16419, Republic of Korea

**Keywords:** combination therapy, dendritic cells, nanoparticle, TLR7/8 agonist, vasculature disruption

## Abstract

This study evaluates the effect of combination of two different treatment regimens for solid tumor therapy: vasculature targeting agent and immune-stimulation. Poly lactide-co-glycolide (PLGA) nanoparticles were synthesized for intracellular delivery of toll-like receptor (TLR) 7/8 agonist—gardiquimod. Spherical and mono-disperse gardiquimod encapsulated PLGA nanoparticles (Gardi-PLGA), approximately 194 nm in size were formulated. Gardi-PLGA induced immune-stimulation, and vasculature disrupting agent (VDA)—5,6-Dimethylxanthenone-4-acetic acid (DMXAA) was used in combination to assessing the influence on bone marrow derived dendritic cells (BMDCs) and B16-F10 melanoma cells. The combination treatment significantly increased the levels of pro-inflammatory cytokines, indicating their activation in BMDCs, while melanoma cells remained viable. Further, mice melanoma model was established, and DMXAA was administered intraperitoneally and Gardi-PLGA was administered via an intra-tumoral injection. The combination treatments strategy significantly inhibited tumor growth as shown by tumor volume analysis, and the survival rate of the mice was found to be 63.6% (n = 11), after 54 days of tumor inoculation. Immunohistochemical findings of tumor sections treated with DMXAA confirmed the *in vivo* vasculature disruption. Thus, the inhibition of tumor growth can be attributed to the synergistic effect of immune stimulation caused by DC activation and vasculature disruption.

## INTRODUCTION

The heterogeneous tumor microenvironment primarily consists of malignant transformed cells, vasculature, lymphatics, and extra-cellular matrix along with a wide repertoire of immune cells such as dendritic cells (DCs), macrophages, fibroblasts, and lymphocytes. While the conventional treatment strategies for solid tumor include surgery, chemotherapy and radiation therapy; immune-cell mediated killing of cancer cells and combination therapy are upcoming approaches. Immunotherapy and combination therapy unlike standalone chemotherapy is less prone to drug resistance, is known to have minimal side-effects [1]. The progression of tumor cells is monitored by immune cells and is called as immune-surveillance [2]. However, tumor cells have not only developed various mechanisms to circumvent immune-surveillance, but can also establish immune-mediated conditions, which expedite tumor development [3]. Targeting the mechanisms adopted by tumor to escape the host immune system and stimulating the suppressed immune system, has led to the development of immunotherapeutic-based treatment. Immunotherapeutic-based approaches include T cells-, DCs-, and natural killer (NK) cells-based therapy [4–6]; small molecule immunomodulatory compounds, protein engineered vaccines [7–9]; and monoclonal antibodies-based therapy [10]. Toll like receptors (TLR) agonists are a class of immuno-stimulatory compounds which are routinely being used as adjuvants for vaccination and also as a candidate for combination therapy in cancer [11]. TLR7 agonist, imiquimod (R837) has shown remarkable results for treatment of superficial basal cell carcinoma, actinic keratosis and perianal and genital warts [12]. TLR7 agonist is known to impart tumor killing activity to plasmacytoid dendritic cells (pDCs) [13]. Recently, TLR8 agonist has also been emphasized for anti-tumor immune response owing to its ability to promote NK-DC cross talk [14, 15].

Gardiquimod (1-(4-amino-2-ethylaminomethylimidazo-[4,5-c]quinolin-1-yl)-2-methylpropan-2-ol) is another TLR7/8 agonist belonging to the class of imidazoquinoline compounds which is known to have higher potency than imiquimod [16]. Gardiquimod has also been known to enhance the efficacy DC-based therapy for melanoma. Combination therapies of TLR-based agonist with other anti-cancer treatment regimens have resulted in encouraging outcomes. TRL7 agonist imiquimod in combination with chemotherapeutic drug paclitaxel has demonstrated improved anti-tumor therapy and immunological memory effect in mouse model [17]. Some studies suggest that combination of chemotherapy with immune-stimulating agents does not influence the anti-tumor efficacy of individual drugs [18]. However, many researchers have illustrated increased anti-tumor effects by using various TLR agonist (TLR4 agonist-LPS, TLR9 agonist-5′-cytosine-phosphate-guanine-3′ (CpG)) in combination with chemotherapeutic drugs [19, 20]. While majority of chemotherapeutic drugs attack the tumor cells and cause direct tumor cell killing, anti-angiogenic drugs and vasculature disrupting agents (VDA) are another class of drugs that inhibit tumor growth by disrupting the tumor vasculature [21]. Anti-angiogenic drugs prevent the formation of new blood vessels and VDAs block tumor vasculature resulting in reduced blood supply to the tumor cells, leading to necrosis. After single treatment, VDAs lead to formation of a necrotic center, while the effect of the drug on the periphery of a solid tumor is not pronounced due to difference in interstitial fluid pressure and vessel architecture [22]. The tumor cells in the periphery also continue to thrive because they receive oxygen and nutrition through blood and they are able to migrate (metastasis) to distant locations in the body. This is a major drawback of VDAs, which needs to be addressed for effective tumor therapy. Since the periphery of the tumor can be readily accessed by the immune cells in the tumor microenvironment, combination of vasculature disruption with immunotherapy anti-tumor effect was explored.

**Scheme 1:**
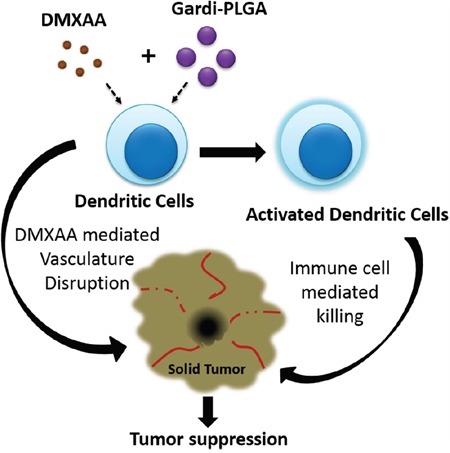
Illustration depicting speculated mode of actions of combination of Gardi-PLGA and DMXAA Gardiquimod encapsulating PLGA nanoparticles in combination with DMXAA is speculated to cause activation of dendritic cells which facilitates immune cell mediated killing of cancer cells. Vasculature disrupting agent DMXAA further aids in inhibition of tumor growth.

The vasculature disrupting agent, 5,6-dimethylxanthenone-4-acetic acid (DMXAA) also known as Vadimezan or ASA404, has shown encouraging results in phase II clinical trial. However, the results were not satisfactory in phase III clinical trial. The molecular target of DMXAA is STING, which is found to be mouse specific and not effective in humans [23]. While research demonstrates human analogue of DMXAA (8-methyl xanthenone-4-acetic acid) has potent anti-tumor activity for human tumors, in this study DMXAA was used as a prototype VDA in mouse model [24]. Another benefit associated with DMXAA is that it is also known to possess immune-stimulatory activity and thus is expected to facilitate the activation of immune cells caused by TLR7/8 agonist gardiquimod [25]. Since the receptors-TLR7 and TLR8 are present on the endosomal membrane of the cells, nanoparticulate formulation was used for delivery of gardiquimod inside the cells. Poly(lactic-co-glycolic acid)-PLGA nanoparticles are known to be non-specifically endocytosed by cells and reach the endosomal compartment where the acidic environment leads to degradation of PLGA matrix, consequently releasing the encapsulated therapeutic agent [26]. Therefore, in this research gardiquimod encapsulated PLGA nanoparticles (Gardi-PLGA) were used as carrier for delivery of gardiquimod and subsequent activation of BMDCs. Further, DMXAA in combination with Gardi-PLGA was tested on BMDCs for analysis of activation status, followed by exploring the effect of combination in mice tumor model. This research work ventures to delineate the combined effect of vasculature disruption therapy by DMXAA and TLR7/8 agonist gardiquimod on tumor treatment (Scheme 1).

## RESULTS AND DISCUSSION

### Characterization of gardiquimod-PLGA nanoparticles and analyzing its efficiency *in vitro*

**Figure 1:**
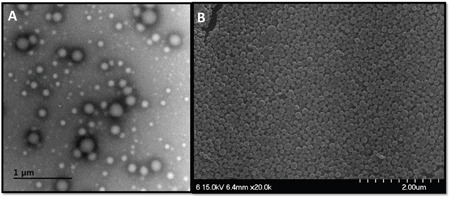
**A.** Transmission electron micrographs (scale bar, 1 μm), and **B**. scanning electron micrographs (scale bar, 2 μm) of the Gardi-PLGA nanoparticles.

The size distribution of gardiquimod-encapsulated PLGA nanoparticles (Gardi-PLGA) was found to be 194 ± 50 nm, with zeta potential of -36.58 mV (Supplementary Figure 1). The encapsulation efficiency of gardiquimod in the particles was 11.1 ± 0.3 μg/mg. TEM and SEM (Figure 1) analyses revealed uniform and spherical morphology of the nanoparticles.

Nano particulate carriers are considered to be more efficient than the free form for delivery of antigens and adjuvants because the drugs contained in nanoparticles are known to impart higher levels of immuno-stimulation than free drugs [27–29]. In order to assess the immuno-stimulatory activity of gardiquimod in free form and nanoparticulate formulation, BMDCs were treated with two concentrations (0.1 and 1 μg/ml) of free gardiquimod and Gardi-PLGA. The gardiquimod concentration in Gardi-PLGA was measured by dissolving the nanoparticles in DMSO, followed by analysis by ultraviolet (UV) spectrometer. The levels of cytokine, TNFα significantly increased in cells treated with nanoparticulate formulation as compared to those of free gardiquimod (Supplementary Figure 2). The effect may be attributed to fact that antigen presenting cells (APCs) are capable of efficient uptake of nanoparticles by endocytosis and phagocytosis. Moreover, PLGA is known to possess adjuvanticity, which could further enhance immuno-stimulation caused by gardiquimod [30].

The uptake of these nanoparticles was assessed by fluorescent imaging of DCs treated with ICG (indocyanine green)-labeled nanoparticles. Punctate-like structures were observed inside the cells, which confirmed the uptake of Gardi-PLGA by DCs (Supplementary Figure 3). Further, it was confirmed that the nanoparticles have been localized in the endosomal compartment inside the cells which is required stimulating TLR7 and TLR8 present in the endosome (Supplementary Figure 4).

### Synergistic activation of BMDCs in the presence of combined DMXAA and Gardi-PLGA

**Figure 2:**
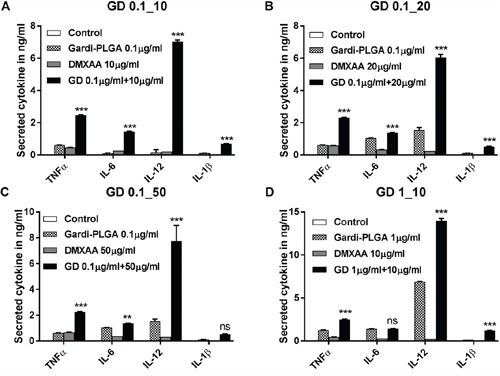
Secretion levels of cytokine (TNFα, IL-6, IL-12 and IL-1β) by BMDCs after treatment with Gardi-PLGA, DMXAA, and their combination at various concentrations **A**. Gardi-PLGA 0.1 μg/mL + DMXAA 10 μg/mL, **B**. Gardi-PLGA 0.1 μg/mL + DMXAA 20 μg/mL, **C**. Gardi-PLGA 0.1 μg/mL + DMXAA 50 μg/mL, and **D**. Gardi-PLGA 1 μg/mL + DMXAA 10 μg/mL. ***p < 0.001 by one-way ANOVA Test, with Tukey's multiple comparison.

**Figure 3:**
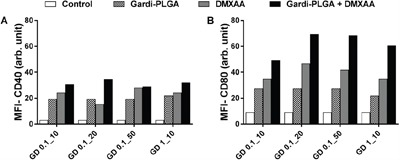
Mean fluorescence intensity (MFI) of A. CD40 and B. CD80 of BMDCs after treatment with Gardi-PLGA, DMXAA, and their combination at various concentrations

BMDCs were treated with varying concentrations of individual drugs (Gardi-PLGA, 0.1 and 10 μg/ml; DMXAA, 10, 20 and 50 μg/ml) and various combination ratios of Gardi-PLGA and DMXAA (GD-0.1_10 μg/ml, GD-0.1_20 μg/ml, GD-0.1_50 μg/ml, GD-1_10 μg/ml). Combined treatment with Gardi-PLGA and DMXAA revealed that GD-0.1_10 and GD-0.1_20 led to significant elevation in cytokine levels (TNFα, IL-6, IL-12, and IL-1β) as compared to control individual drugs (Figure 2A and 2B). TNFα, IL-6, and IL-12 were found to be significantly enhanced when GD-0.1_50 was used. TNFα, IL-12, and IL-1β levels increased considerably at GD-1_10 ratio (Figure 2C and 2D). Interestingly, the effect of combination treatment is not only additive but also synergistic when compared with individual drugs for most of the combination ratios. Further, levels of maturation markers (CD40 and CD80) were analyzed to determine the level of immune stimulation. The combination treatment demonstrated enhanced expression of maturation markers as compared to control and individual drugs (Figure 3), indicating an enhanced immune stimulation in the presence of combination. The enhanced immune-stimulatory effect in the presence of combination treatment could be attributed to the fact that the immune-stimulatory response of Gardi-PLGA and DMXAA is facilitated by different pathways inside a cell. It is known that MyD88 pathway and nuclear factor κB (NF-κB)–dependent gene expression are associated with the immune-stimulatory effect of TLR agonist—gardiquimod while the target of DMXAA is TANK-binding kinase 1 (TBK1)–interferon (IFN) regulatory factor 3 (IRF-3), a non-MyD88 pathway with minimal NF-κB–dependent gene expression [31, 32]. Therefore, when the cells are treated with the combination of these two drugs, two pathways are triggered inside the cell, which could possibly be the reason for synergistic activation of DCs and be speculated to enhance T cell response *in vivo*.

### *In vitro* and *in vivo* anti-tumor effect of combination of DMXAA and Gardi-PLGA

In order to assess the cytotoxic activity of the two drugs, B-16-F10 melanoma cells were treated with Gardi-PLGA, DMXAA, and their combination at various concentrations. The cell proliferation data observed after 24 and 48 hours showed no cytotoxicity in the presence of the two drugs individually as well as in combination (Supplementary Figure 5). Thus, unlike chemotherapeutic agents, both Gardi-PLGA and DMXAA are not cytotoxic towards melanoma cells.

**Figure 4:**
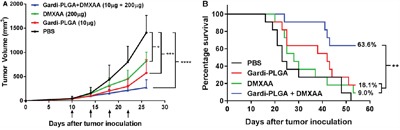
**A**. Tumor volume (mm^3^) with respect to day 1 until day 27 in C57BL/6 mice immunized with Gardi-PLGA, DMXAA, and their combination. Injection time points are indicated by black arrows. Significant inhibition in tumor growth was observed after treatment with combination. *p < 0.05, ***p < 0.001, ****p < 0.0001 by one-way ANOVA Test, Bonferroni's post-test, n = 8. **B**. Kaplan Meier survival plot of immunized mice is shown. **p = 0.0024, n = 11.

The combination treatment was tested *in vivo* in a mouse melanoma model. Tumor growth was inhibited in the presence of individual drugs as wells as combination of Gardi-PLGA and DMXAA until 22 days after tumor inoculation. However, after 26 days, in case of individual drugs (Gardi-PLGA only and DMXAA only), the tumor continued to grow, while the growth was significantly diminished for combination drug-treated group (Figure 4A). The combination also led to significant increase in the survival percentage of mice (63.6%) as compared to individual treatment of Gardi-PLGA (18.1%), DMXAA (9%) and control PBS group, 54 days after tumor inoculation (Figure 4B). These observations suggest that individual drug treatment with standalone vasculature disrupting agent or immune-stimulatory TLR7/8 agent is capable of illustrating partial anti-tumor effect. However the combination of the two agents at same dose is capable of producing pronounced anti-tumor effects.

The limited effect of VDA could possibly be attributable to its inability to disrupt the periphery of the solid tumor, leading to the presence of a viable rim which sustains the tumor [33]. The immunostimulatory effect of standalone gardiquimod is speculated to be non-specific owing to the absence of tumor antigen, which diminishes the anti-tumor response. The combination of both the drugs is speculated to cause massive tumor cell necrosis at tumor center (due to DMXAA), which leads to release of necrotic particles which can act as tumor antigen, that imparts specificity to gardiquimod-mediated immune response, thus leading to prominent inhibition in tumor growth and distinct survival of mice.

### Analysis of tumor sections after treatment with DMXAA and Gardi-PLGA

**Figure 5:**
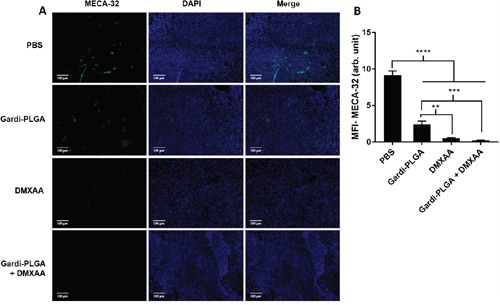
**A**. Immunohistochemical images of tumor sections at day 14 after tumor inoculation for Gardi-PLGA, DMXAA, and their combination labelled with (left to right) endothelial marker (MECA-32, green), nuclear stain (DAPI, blue), and merged. **B**. Mean fluorescence intensity (MFI) of MECA-32 (n = 3 tumor sections, average of 10 different regions for each sample). Significant decrease in presence of endothelial cell marker confirmed vasculature disruption in combination treated groups. ****p < 0.0001, ***p < 0.001, **p < 0.01 by one-way ANOVA Test, with Tukey's multiple comparison.

**Figure 6:**
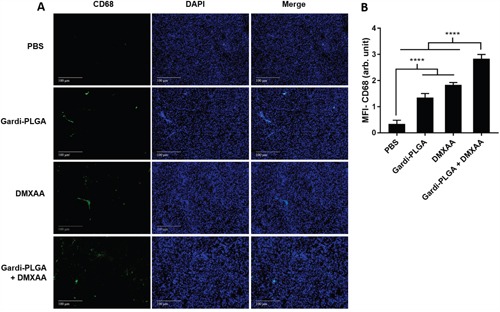
**A**. Immunohistochemical images of tumor sections at day 14 after tumor inoculation for Gardi-PLGA, DMXAA, and their combination labelled with (left to right) macrophage marker (CD68, red), nuclear stain (DAPI, blue), and merged. **B**. Mean fluorescence intensity (MFI) of CD68 (n = 3 tumor sections, average of 10 different regions for each sample). Significant increases in presence of macrophage marker are shown in the treated groups. ****p < 0.0001 by one-way ANOVA Test, with Tukey's multiple comparison.

**Figure 7:**
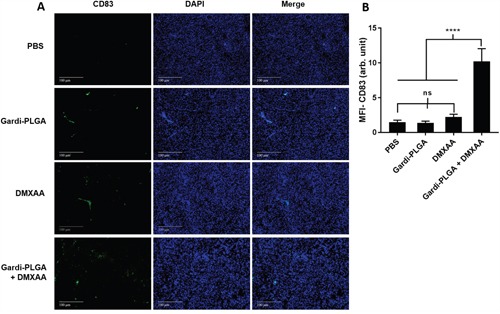
**A**. Immunohistochemical images of tumor sections at day 14 after tumor inoculation for Gardi-PLGA, DMXAA, and their combination labelled with (left to right) dendritic cells marker (CD83, green), nuclear stain (DAPI, blue), and merged. **B**. Mean fluorescence intensity (MFI) of CD83 (n = 3 tumor sections, average of 10 different regions for each sample). Significant increase of dendritic cell infiltration is shown only in combination treated group. ****p < 0.0001 by one-way ANOVA Test, with Tukey's multiple comparison.

**Figure 8:**
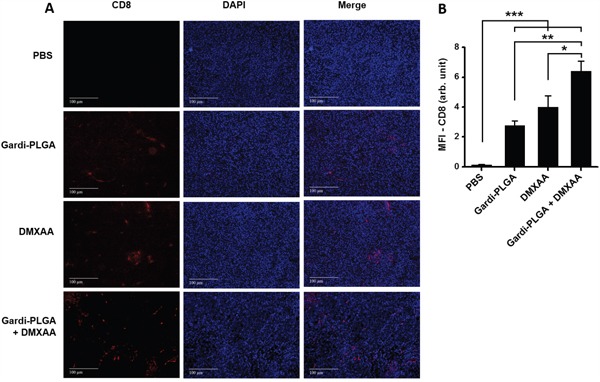
**A**. Immunohistochemical images of tumor sections at day 14 after tumor inoculation for various treatment groups labelled with (left to right) T-cell marker (CD8, red), nuclear stain (DAPI, blue), and merged. **B**. Mean fluorescence intensity (MFI) of CD8 (n = 3 tumor sections, average of 10 different regions for each sample). There was significant increases of CD8 T cell infiltration in the DMXAA- and Gardi-PLGA-treated groups compared to PBS group, and further significant increase was observed in combination treated group. *p < 0.05, **p < 0.01, and ***p < 0.001 by one-way ANOVA Test, with Tukey's multiple comparison.

The histological findings of tumor sections for endothelial cell marker MECA-32 were performed to assess the vasculature disruption in tumor. Clusters of endothelial cells indicating intact blood vessels were observed in PBS- and Gardi-PLGA treated groups. However the signal drastically diminished in DMXAA and combination treated groups, indicating vasculature disruption (Figure 5). The diminished signal intensity of MECA-32 after individual and combination treatment of DMXXA suggested possible cytoskeletal rearrangement of endothelial cells and/or apoptosis. Endothelial cell apoptosis leads to cascade of events including high interstitial pressure, reduced blood flow, increased viscosity, and red blood cell stacking [33]. The signal for endothelial cells is also expected to be diminished owing to blockage caused by red blood cells after DMXAA treatment. In case of Gardi-PLGA treated tumors, the decreased number of endothelial cells may be due to TNFα (secreted by activated DCs) mediated vasculature disruption [34]. The shutdown of blood flow in tumor would cause oxygen and nutrients deprivation in the tumor cells, thus leading to massive necrosis. This could further lead to release of large number of tumor antigens and damage associated molecular patterns (DAMPs), causing activation of resident immature DCs. The tumor antigen released can further specify and direct the immune response generated by Gardi-PLGA-activated DCs. The released tumor antigen is also expected to reach systemic circulation and is speculated to keep a check on tumor metastasis at distant sites by causing immuno-activation as previously reported in a similar study [17]. The activation of tumor-resident APCs (macrophages and DCs) was confirmed by immunohistochemistry (IHC) by using specific markers for macrophages (CD68) and activated DCs (CD83). The macrophage population was significantly enhanced in tumors treated with both Gardi-PLGA and DMXAA individually as well as in combination; however the level of significance was considerably higher for combination treatment (Figure 6). It is well established that the immunostimulatory activity induced by gardiquimod is due to TLR7/8 stimulation. DMXAA is also known to have immune-stimulatory effect which could lead an increase in the number of macrophages in the tumor [25, 32]. It is reported to cause immune-stimulation via activation of interferon response factor-3 signaling and is also known to contribute to anti-tumor immune response by changing the phenotype of macrophages [31, 35]. This suggests the possible immune-stimulation by DMXAA which augments the overall anti-tumor immune response. The treatment with individual drugs was not able to improve the number of DCs in tumor sections but combination treatment demonstrated drastic increase in the number of DCs (Figure 7). The infiltration of CD8+ T cells in tumors can be also increased by DC activation with DMXAA or Gardi-PLGA, in which CD8+ T cells are known to have cytolytic activity against tumor cells. The infiltration frequency of CD8+ T cells in tumor sites was also confirmed by the IHC measurement for CD8+ T cells in the tumor tissues, showing significant increase of CD8+ T cell infiltration in tumors for both the DMXAA- and Gardi-PLGA-treated groups (Figure 8). Further, in combination treated group, the amount of infiltrated CD8+ T cells was significantly increased compared to DMXAA- or Gardi-PLGA-treated group. This indicates the doses of DMXAA and Gardi-PLGA used individually were insufficient to cause immunostimulatory effect on DCs; however, combination treatment of the drugs led to synergistic activation (confirmed by *in vitro* studies) that subsequently led to favorable anti-tumor immune response (Supplementary Figure 6).

## MATERIALS AND METHODS

### Preparation of Gardi-PLGA and DMXAA solution

5 mg of gardiquimod (Sigma, St. Louis, MO, USA) with 60 mg of PLGA (Sigma) in 2 ml of dichloromythelene (DCM) was added to 10 ml of 2.5% poly (vinylalcohol) and sonicated for 2 minutes in a probe sonicator. DCM was allowed to evaporate overnight, by stirring the emulsion at 600 rpm using a magnetic stirrer. The nanoparticle suspension was centrifuged at 4,000 rpm for 3 min and the pellet was discarded to remove large particles. The supernatant was centrifuged at 13,000 rpm for 15 minutes followed by dispersion in 30 ml of distilled water. This process was repeated for three times. Nanoparticles were freeze-dried and stored until further use. For preparation of indocyanine green (ICG, Dongindang Pharm., Siheung, Korea)-encapsulated PLGA nanoparticles, 5 mg of ICG was added in the first step, and followed the same procedure as mentioned above. DMXAA solution was prepared by dissolving 5 mg of DMXAA (Sigma) in 1 ml of 5% sodium bicarbonate (Sigma) solution.

### Characterization of Gardi-PLGA

The morphology of nanoparticles was observed by using scanning electron microscope (SEM, S4700; Hitachi, Germany) and transmission electron microscope (TEM, Tecnai G2 F30; FEI, Hillsboro, OR, USA). Nanoparticle size and size distribution was measured using electrophoretic light scattering (ELS Z; Otsuka Electronics, Osaka, Japan). For determination of encapsulation efficiency, 5 mg of nanoparticles was dissolved in 1 ml of dimethyl sulfoxide (DMSO) and absorbance was measured at 332 nm using S-3100 spectrophotometer (Scinco, Seoul, Korea). The concentration of gardiquimod in Gardi-PLGA was analyzed from the standard curve of gardiquimod in DMSO at 332 nm.

### *In vitro* viability assay

B-16-F10 murine melanoma cell line (American Type Culture Collection-ATCC) was tested for *in vitro* viability after treatment with Gardi-PLGA (0.1 and 1 μg/ml of gardiquimod), DMXAA (10, 20, 50 μg/ml), and combination of both at various concentrations. MTS assay for analysis of mitochondrial activity was used as a measure of cell viability. 1 × 10^4^ cells in 100 μl Dulbecco's Modified Eagle's medium supplemented with 10% fetal bovine serum and an antibiotic–antimycotic mixture (Invitrogen-Gibco, Carlsbad, CA, USA) were seeded per well in a 96 well plate (Corning Costar, Cambridge, MA, USA). Drug sample (100 μl) dispersed in cell culture media was added and incubated for 24 and 48 hours at 37°C and 5% CO_2_/95% humidified air. After incubation, 20 μl of MTS solution-Cell Titer 96 Aqueous One Solution kit (Promega, Madison, WI, USA) was added to each well and incubated for 2 hours. The absorbance of the samples was read at 490 nm (VersaMax, Molecular Devices, Sunnyvale, CA, USA) and was normalized with respect to absorbance of untreated cells.

### *In vitro* BMDC activation and maturation

Bone marrow derived dendritic cells (BMDCs) were isolated from mouse bone marrow as reported previously [36]. BMDCs (1 × 10^6^) suspended in 1 ml of RPMI media supplemented with 10% FBS and 1% antibiotic-antimycotic solution were seeded per well in a 6 well plate (Corning Costar). Cells were treated with 1 ml of drug solution and incubated for 24 hours at 37°C and 5% CO_2_/95% humidified air. The plates were centrifuged at 1,500 rpm for 5 minutes and the supernatant was collected and was analyzed for various cytokines (IL-1β, IL-6, IL-12, and TNFα) using ELISA kit (BD Biosciences, Franklin Lakes, New Jersey). For analysis of upregulation of maturation marker after incubating with drug samples for 24 hours, the cells were harvested and fixed using 4% paraformaldehyde. They were treated with fluorescence labeled marker antibody FITC-CD40 (BD PharMingen, San Diego, CA, USA) and PE-CD80 (eBioscience, San Diego, CA, USA) by incubating at 4°C for 30 minutes followed by washing with PBS twice. The cells (10^4^) were then analyzed for fluorescence using flow cytometer (Attune Acoustic Focusing Cytometer, Life Technologies, California, USA).

### Nanoparticle uptake by cells and fluorescence microscopic imaging of cells

DC2.4 cells were obtained from Dr. Kenneth L. Rock (Dana-Farber Cancer Institute, Boston, MA, USA) and cultured in RPMI 1640 medium (Gibco-BRL, Grand Island, NA, USA) supplemented with 10% heat-inactivated fetal bovine serum (FBS, Gibco), 1% antibiotic-antimycotic (Gibco) at 37°C in humidified air containing 5% CO_2_. To determine the intracellular delivery of nanoparticles, DC2.4 cells were incubated with Gardi-PLGA (0.1 μg of gardiquimod) and ICG-encapsulated PLGA nanoparticles in 8 well μ-slide (Ibidi Integrated Biodiagnostics, Munich, Germany) at a density of 1 × 10^4^ cells/well, at 37°C for 24 hours. Unbound nanoparticles were removed by washing with phosphate buffered saline (PBS, Gibco). The cells were fixed with 4% paraformaldehyde solution for 15 min at room temperature, and treated with 4.6-diamino-2-phenylindoledichydrochloride (DAPI, Sigma, St. louis, MO, USA) in PBS for 10 min. Fluorescence microscopy was performed on a Deltavision RT (Applied Precision Technologies, Issaquah, WA, USA). To detect intracellular localization of ICG-Gardi-PLGA nanoparticles, cells were stained with Lysotracker Blue (Invitrogen) for 15 minutes and fluorescence images were obtained after 1 h incubation.

### Mice and cell lines

5-6 weeks-old female C57BL/6 (H-2b) mice were purchased from KOATECH (Pyeongtaek, Korea). The mice were maintained under pathogen-free conditions. All experiments employing mice were performed in accordance with the Korean NIH guidelines for care and use of laboratory animals. B16-F10 murine melanoma cell line was purchased from ATCC, and it was allowed to grow in DMEM supplemented with 10% FBS and 1% antibiotic-antimycotic solution at 37°C and 5% CO_2_/95% humidified air.

### Evaluation of *in vivo* antitumor activity

B16-F10 melanoma cells (1 × 10^5^) were inoculated into the right flank of 5-6 weeks old C57BL/6 mice. After 10 days of tumor implantation, animals with an average tumor diameter of 4-6 mm were selected. These mice were divided into 4 treatment groups (n = 8 for tumor volume analysis and n = 11 for survival analysis). Gardi-PLGA nanoparticles in 50 μl of PBS were administered by intratumoral injection and DMXAA solution (100 μl) was administered by intra-peritoneal injection. The drug treatment was started from day 10 after tumor implantation. Total 4 injections were given (days 10, 14, 18 and 22). The tumor diameters were measured till 26^th^ day after tumor implantation using a sliding caliper. Tumor volume was calculated using the following formula: tumor volume (mm^3^) = length x (width)^2^/2. After 8 weeks mice were humanely euthanized.

### Evaluation of vasculature disruption and presence of immune cells in *ex vivo* tumor tissues using immunohistochemistry

The xenograft tissues (n = 3 for each group) were fixed with 10% formalin and embedded in paraffin. The paraffinized tissues were cut into 2-μm sections and placed on frontier micro slide glass. The sections were deparaffinized by 99.9% xylene, following 100%, 95%, 70% ethanol and finally distilled water. The sections were fixed with 4% paraformaldehyde, permeabilized in 0.2% trition in PBS, then blocked with 5% NGS (normal goat serum) for 2 hour, followed by incubating with a primary antibody, which was used at a final concentration of 5-10 μg/ml in 2% NGS and included: anti-mouse endothelial cell (MECA-32, BioLegend, California, USA), mouse anti-rat CD68 (AbD Serotec, Kidlington, UK), anti-CD83 (Michel-19, BioLegend), aand anti-mouse CD8 (BioLegend, California, USA). Sections were incubated overnight with primary antibody at 4°C and then incubated with appropriate horseradish peroxidase-conjugated secondary antibody (Santa Cruz Biotechnology, Santa Cruz, CA, USA) after washing with 0.1% Trition in PBS. The sections were finally mounted using DAPI Fluoromount-G (SouthernBiotech, Birmingham, USA). Fluorescent images were captured by Olympus microscope BX53 using MetaVue software under the same conditions (10 or 1,000 ms exposure time, high & low limits, and scaling) and the signal intensity was analyzed with the threshold images that excluded the background signal.

### Statistical analysis

For analyzing the statistical difference between two groups one-way ANOVA with multiple comparisons was used. For analyzing statistical significance in survival data log-rank (ManteleCox) test was used and p value <0.05 was considered statistically significant. All values are expressed as mean ± standard deviation. GraphPad Prism (San Diego, CA, USA) was used for all statistical analysis.

## CONCLUSION

Both Gardi-PLGA and DMXAA did not possess direct tumor killing activity as confirmed by viability assay on B-16-F10 melanoma cells. Thus, the tumor inhibition observed *in vivo* can be attributed to the immuno-stimulatory effect of combined Gardi-PLGA and DMXAA along with vasculature disrupting effect of DMXAA. The synergy in the combination of DMXAA and Gardi-PLGA mainly works at two levels. Firstly, synergistic immuno-stimulatory effect is observed in DCs. Secondly, tumor inhibition is speculated to be achieved by targeting the tumor center by vasculature disruption and tumor periphery mainly by the immune cells. If a human analogue of DMXAA [24] or any other vasculature disrupting agent is successfully identified, its combination with immune-stimulating agent such as TLR agonists seems to have remarkable potential for treatment of solid tumors.

## SUPPLEMENTARY FIGURES



## References

[1] Blagosklonny MV (2005). How Avastin potentiates chemotherapeutic drugs: action and reaction in antiangiogenic therapy. Cancer Biol Ther.

[2] Kim R, Emi M, Tanabe K (2007). Cancer immunoediting from immune surveillance to immune escape. Immunology.

[3] Schreiber RD, Old LJ, Smyth MJ (2011). Cancer Immunoediting: Integrating Immunity's Roles in Cancer Suppression and Promotion. Science.

[4] Cheng M, Chen Y, Xiao W, Sun R, Tian Z (2013). NK cell-based immunotherapy for malignant diseases. Cell Mol Immunol.

[5] Palucka K, Banchereau J (2012). Cancer immunotherapy via dendritic cells. Nat Rev Cancer.

[6] Houot R, Schultz LM, Marabelle A, Kohrt H (2015). T-cell–based Immunotherapy: Adoptive Cell Transfer and Checkpoint Inhibition. Cancer Immunol Res.

[7] Nicholas C, Lesinski GB (2011). Immunomodulatory cytokines as therapeutic agents for melanoma. Immunotherapy.

[8] Mahoney KM, Rennert PD, Freeman GJ (2015). Combination cancer immunotherapy and new immunomodulatory targets. Nat Rev Drug Discov.

[9] Talmadge JE (2016). Genetically Engineered Multivalent Proteins for Targeted Immunotherapy. Clin Cancer Res.

[10] Scott AM, Wolchok JD, Old LJ (2012). Antibody therapy of cancer. Nat Rev Cancer.

[11] Dunne A, Marshall NA, Mills KHG (2011). TLR based therapeutics. Curr Opin Pharmacol.

[12] Vacchelli E, Galluzzi L, Eggermont A, Fridman WH, Galon J, Sautès-Fridman C, Tartour E, Zitvogel L, Kroemer G (2012). Trial watch: FDA-approved Toll-like receptor agonists for cancer therapy. Oncoimmunology.

[13] Stary G (2007). Tumoricidal activity of TLR7/8-activated inflammatory dendritic cells. J Exp Med.

[14] Zhou Z, Yu X, Zhang J, Tian Z, Zhang C (2015). TLR7/8 agonists promote NK–DC cross-talk to enhance NK cell anti-tumor effects in hepatocellular carcinoma. Cancer Letters.

[15] Schon MP, Schon M (2008). TLR7 and TLR8 as targets in cancer therapy. Oncogene.

[16] Ma F, Zhang J, Zhang J, Zhang C (2010). The TLR7 agonists imiquimod and gardiquimod improve DC-based immunotherapy for melanoma in mice. Cell Mol Immunol.

[17] Seth A, Heo MB, Lim YT (2014). Poly (γ-glutamic acid) based combination of water-insoluble paclitaxel and TLR7 agonist for chemo-immunotherapy. Biomaterials.

[18] Qu X, Felder MAR, Perez Horta Z, Sondel PM, Rakhmilevich AL (2013). Antitumor effects of anti-CD40/CpG immunotherapy combined with gemcitabine or 5-fluorouracil chemotherapy in the B16 melanoma model. Int Immunopharmacol.

[19] Roy A, Singh MS, Upadhyay P, Bhaskar S (2013). Nanoparticle mediated co-delivery of paclitaxel and a TLR-4 agonist results in tumor regression and enhanced immune response in the tumor microenvironment of a mouse model. Int J Pharm.

[20] Vicari A, Luu R, Zhang N, Patel S, Makinen S, Hanson D, Weeratna R, Krieg A (2009). Paclitaxel reduces regulatory T cell numbers and inhibitory function and enhances the anti-tumor effects of the TLR9 agonist PF-3512676 in the mouse. Cancer Immunol Immunother.

[21] Thorpe PE (2004). Vascular Targeting Agents as Cancer Therapeutics. Clin Cancer Res.

[22] Hill SA, Chaplin DJ, Lewis G, Tozer GM (2002). Schedule dependence of combretastatin A4 phosphate in transplanted and spontaneous tumour models. Int J Cancer.

[23] Conlon J, Burdette DL, Sharma S, Bhat N, Thompson M, Jiang Z, Rathinam VAK, Monks B, Jin T, Xiao TS, Vogel SN, Vance RE, Fitzgerald KA (2013). Mouse, but not Human STING, Binds and Signals in Response to the Vascular Disrupting Agent 5,6-Dimethylxanthenone-4-Acetic Acid. J Immunol.

[24] Tijono SM, Guo K, Henare K, Palmer BD, Wang LCS, Albelda SM, Ching LM (2013). Identification of human-selective analogues of the vascular-disrupting agent 5,6-dimethylxanthenone-4-acetic acid (DMXAA). Br J Cancer.

[25] Zhou S, Kestell P, Baguley BC, Paxton JW (2002). 5,6-Dimethylxanthenone-4-Acetic Acid (DMXAA): a New Biological Response Modifier for Cancer Therapy. Invest New Drugs.

[26] PANYAM J, ZHOU W-Z, PRABHA S, SAHOO SK, LABHASETWAR V (2002). Rapid endo-lysosomal escape of poly(dl-lactide-co-glycolide) nanoparticles: implications for drug and gene delivery. FASEB J.

[27] Gregory AE, Williamson D, Titball R (2013). Vaccine delivery using nanoparticles. Front Cell Infect Microbiol.

[28] Zhao L, Seth A, Wibowo N, Zhao C-X, Mitter N, Yu C, Middelberg APJ (2014). Nanoparticle vaccines. Vaccine.

[29] Seth A, Oh D-B, Lim YT (2015). Nanomaterials for enhanced immunity as an innovative paradigm in nanomedicine. Nanomedicine.

[30] Akagi T, Baba M, Akashi M, Kunugi S, Yamaoka T (2012). Biodegradable Nanoparticles as Vaccine Adjuvants and Delivery Systems: Regulation of Immune Responses by Nanoparticle-Based Vaccine. Polymers in Nanomedicine.

[31] Roberts ZJ, Goutagny N, Perera P-Y, Kato H, Kumar H, Kawai T, Akira S, Savan R, van Echo D, Fitzgerald KA, Young HA, Ching L-M, Vogel SN (2007). The chemotherapeutic agent DMXAA potently and specifically activates the TBK1–IRF-3 signaling axis. J Exp Med.

[32] Wallace A, LaRosa DF, Kapoor V, Sun J, Cheng G, Jassar A, Blouin A, Ching L-M, Albelda SM (2007). The Vascular Disrupting Agent, DMXAA, Directly Activates Dendritic Cells through a MyD88-Independent Mechanism and Generates Antitumor Cytotoxic T Lymphocytes. Cancer Res.

[33] Tozer GM, Kanthou C, Baguley BC (2005). Disrupting tumour blood vessels. Nat Rev Cancer.

[34] Ruegg C, Yilmaz A, Bieler G, Bamat J, Chaubert P, Lejeune FJ (1998). Evidence for the involvement of endotheliai cell integrin αvβ3 in the disruption of the tumor vascuiature induced by TNF and IFN-[gamma]. Nat Med.

[35] Fridlender ZG, Jassar A, Mishalian I, Wang LC, Kapoor V, Cheng G, Sun J, Singhal S, Levy L, Albelda SM (2013). Using macrophage activation to augment immunotherapy of established tumours. Br J Cancer.

[36] Kim JH, Noh Y-W, Heo MB, Cho MY, Lim YT (2012). Multifunctional Hybrid Nanoconjugates for Efficient In Vivo Delivery of Immunomodulating Oligonucleotides and Enhanced Antitumor Immunity. Angew Chem Int Ed Engl.

