# Supplementary material for: Combining vasculature disrupting agent and toll-like receptor 7/8 agonist for cancer therapy
Source: Oncotarget. 2016 Dec 27;8(3):5371–81. doi: 10.18632/oncotarget.14260 (PMC5354915; doi:10.18632/oncotarget.14260)
Supplement: Supplementary file 1 [file oncotarget-08-5371-s001.pdf]

Combining vasculature disrupting agent and toll-like receptor 7/8 agonist for cancer therapy

SUPPLEMENTARY FIGURES

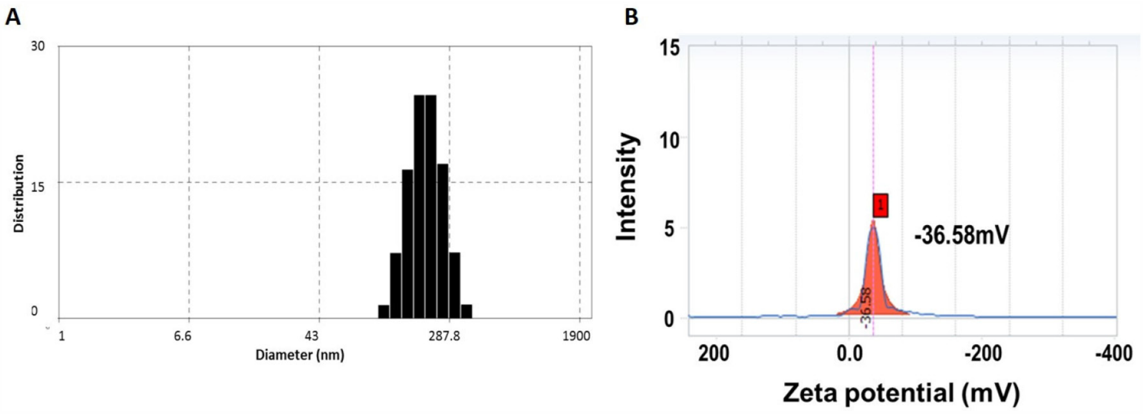

Supplementary Figure 1: A. Particle size distribution and B. zeta potential of Gardi-PLGA nanoparticles measured by dynamic light scattering.

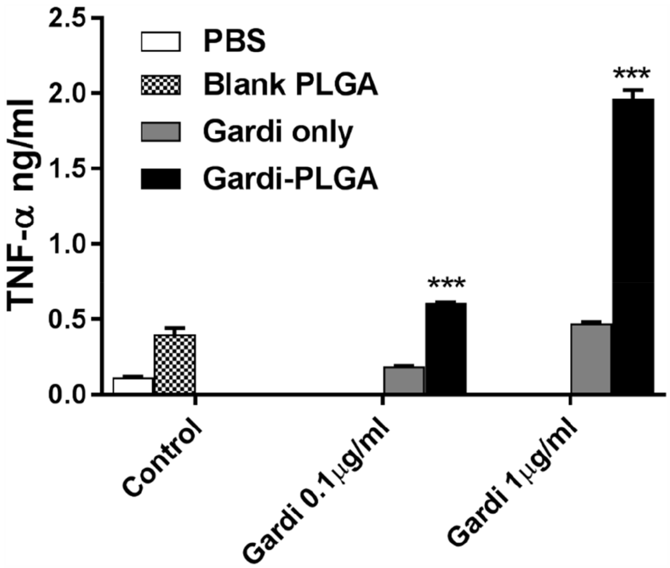

Supplementary Figure 2: TNFα cytokine secretion levels of BMDCs after treatment with blank PLGA nanoparticles (Blank PLGA), gardiquimod only, and gardiquimod-encapsulated PLGA nanoparticles (Gardi-PLGA). ***p<0.001 by one-way ANOVA Test, Bonferroni's post test.

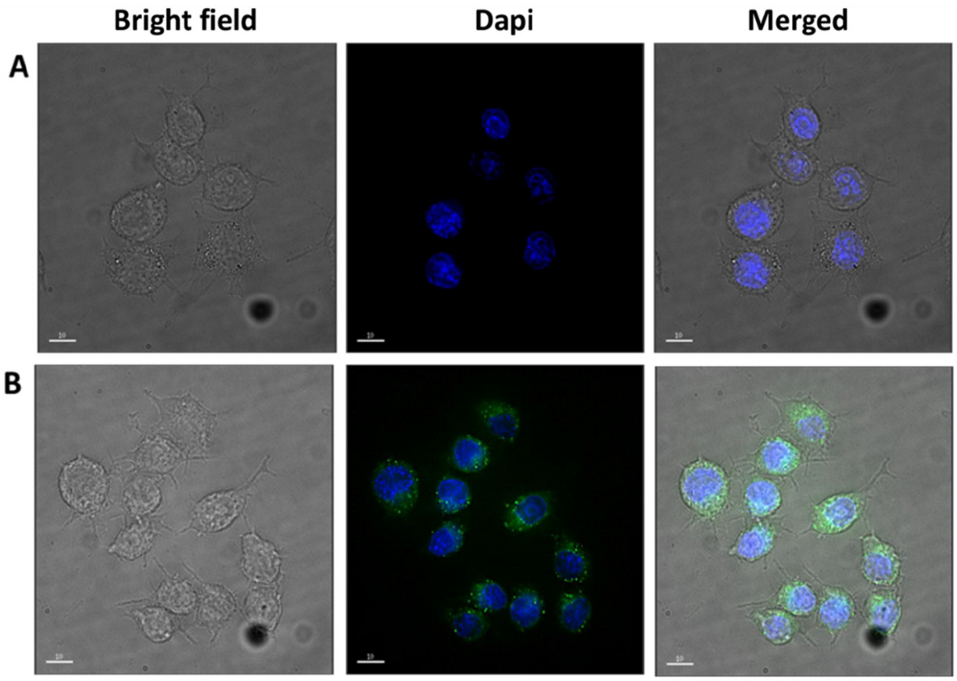

Supplementary Figure 3: Nanoparticle uptake by cells was confirmed by fluorescence imaging of DC2.4 cells after treatment with ICG- and gardiquimod-encapsulated PLGA nanoparticles (ICG-Gardi-PLGA). Bright field, nuclear stained (dapi, blue), and merged images (left to right) of **A.** control cells and **B.** ICG-labelled nanoparticles (green)-treated cells.

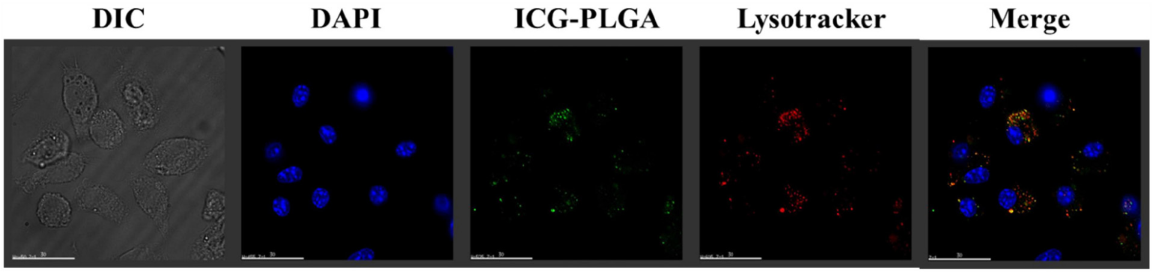

Supplementary Figure 4: Localization of ICG- and gardiquimod-encapsulated PLGA nanoparticles (ICG-Gardi-PLGA) after intracellular delivery into DCs. The ICG-Gardi-PLGA NPs were co-localized in endosome labeled with Lysotracker Blue fluorophore.

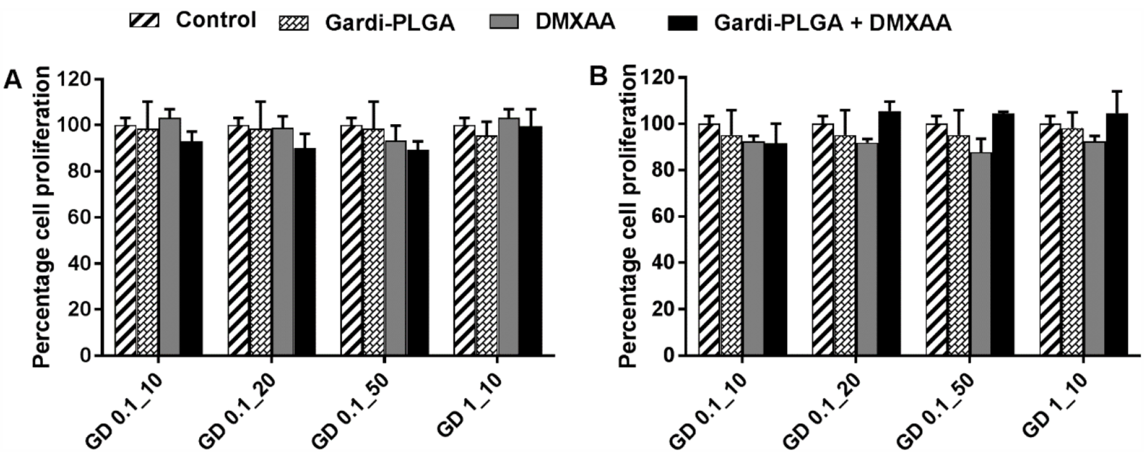

Supplementary Figure 5: Percentage proliferation of B-16-F10 melanoma cells after A. 24 hours and **B.** 48 hours of treatment with Gardi-PLGA, DMXAA, and combination at various concentrations.

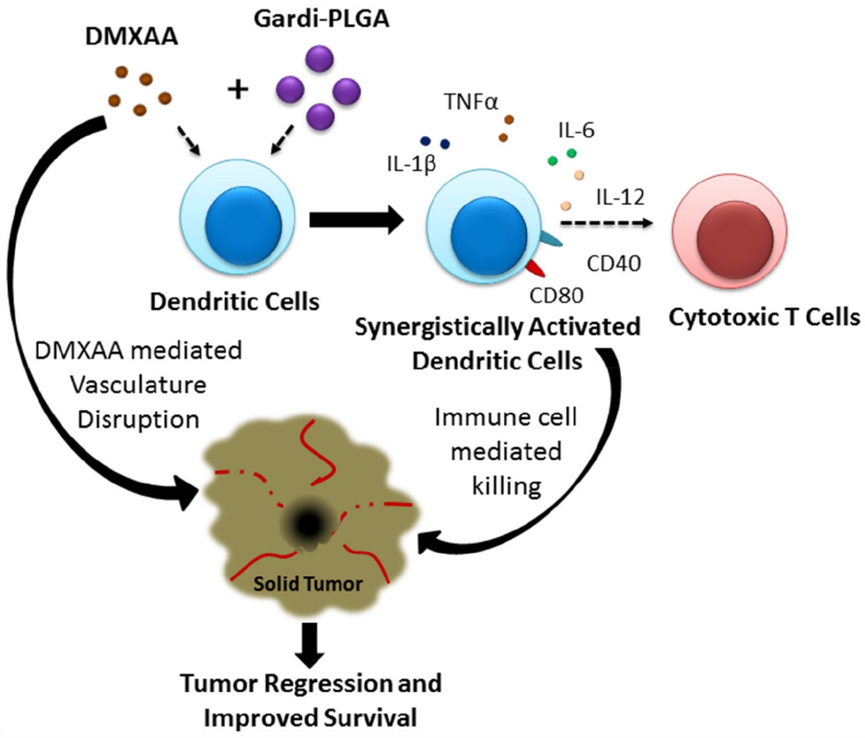

Supplementary Figure 6: DMXAA causes blockage of tumor blood vessel leading to formation of necrotic center in tumor mass. DCs are synergistically activated by DMXAA and gardiquimod-encapsulated in PLGA nanoparticles (Gardi-PLGA). Synergistic activation was confirmed *in vitro* by analysis of secretion levels of pro-inflammatory cytokines (IL-6, TNFα, IL-12 and IL-1β) and upregulation of maturation markers (CD40 and CD80). The activated DCs cause direct killing or cytotoxic T cell-mediated killing of tumor cells. This immune cell-mediated killing also keeps a check on the spared tumor cells present on the periphery. The combined effect of vasculature disruption and immuno-stimulation leads to improved inhibition in tumor growth and survival in mice.
